# Comparison of different pain management strategies during the perioperative period of esophageal squamous cell carcinoma: a retrospective cohort study

**DOI:** 10.1186/s13741-024-00488-3

**Published:** 2025-01-06

**Authors:** Yan Ma, Haiyan Wu, Xinqi Wei, Ying Yang, Zhiyun Xu, Yunyun Chen

**Affiliations:** Department of Thoracic Surgery, The Affiliated Huaian No. 1, People’s Hospital of Nanjing Medical University , Huaian, 223300 China

**Keywords:** Esophageal squamous cell carcinoma, Perioperative pain management, “5 + nursing” pain management strategy, Postoperative complications

## Abstract

**Objective:**

This retrospective cohort study aims to evaluate and compare different postoperative pain management strategies for esophageal squamous cell carcinoma (ESCC), in order to provide scientific evidence for clinical practice and decision-making.

**Methods:**

A total of 274 ESCC patients who underwent surgery at the Affiliated Huai’an No. 1 People’s Hospital of Nanjing Medical University were included in the study. Of these, 127 received conventional nursing decisions for postoperative pain management, and 147 received the “5 + nursing” postoperative pain management strategy. The main observation indicators of both groups included postoperative pain score, analgesic dosage, postoperative analgesic side effects, and length of hospital stays.

**Results:**

The “5 + nursing” postoperative pain management group showed significantly lower postoperative pain score and significantly shorter length of hospital stays than the conventional nursing group. There was no significant difference in postoperative analgesic side effects between the two groups. Multiple logistic regression analysis showed that the postoperative pain score is an independent risk factor for predicting postoperative arrhythmias in ESCC patients. When the daily average dose of opioids used postoperatively was between 37.5 and 50 mg, the patient’s postoperative pain score dropped the fastest.

**Conclusion:**

The “5 + nursing” pain management strategy can effectively reduce the degree of postoperative pain and shorten the length of hospital stays, improving patient’s quality of life. Our research emphasizes the importance of opioids in postoperative pain management, as well as the need for individualized perioperative pain management strategies.

## Introduction

Esophageal squamous cell carcinoma (ESCC) is a rapidly rising digestive system tumor with increasing incidence rates worldwide (Xu et al. 2019; Al-Haddad et al. 2023; Okamura et al. 2023). Clinical surgery remains pivotal in enhancing patient survival rates and improving treatment outcomes (Ma et al. 2023; Teranishi et al. 2023; Li et al. 2023; Patel et al. 2023). Nevertheless, patients undergoing esophageal cancer surgery often endure significant postoperative pain, presenting substantial challenges to both their quality of life and the recovery process (Liang et al. 2023; Sheng et al. 2022; Oh et al. 2017). The management of perioperative pain in ESCC is thus a critical focus for clinical healthcare practitioners (Yoon et al. 2021; Ma et al. 2018).

Postoperative pain exerts profound effects beyond mere discomfort, impacting the healing trajectory and even influencing long-term patient outcomes (Luo et al. 2022; Blazeby et al. 2005). Insufficient pain control in ESCC patients can lead to a cascade of detrimental effects, including prolonged hospitalization, increased risk of infection, and potential cardiovascular complications such as arrhythmias (Yano et al. 2012; Le et al. 2023). Such complications may arise due to the physiological stress imposed by unmanaged pain, which can induce a stress response characterized by increased sympathetic nervous system activity. This heightened stress response not only is a risk factor for immediate postoperative issues but also can contribute to the weakening of the patient’s physiological status, thereby potentially impinging upon long-term survival prospects.

The strategy for perioperative pain management in ESCC must therefore be comprehensive, targeting effective pain alleviation while safeguarding patient recovery and safety (Scheffer et al. 2017; Cronin et al. 2022). Strategies employed currently encompass a range of pharmacological interventions, physical therapies, nerve block techniques, and psychological support mechanisms (Urits et al. 2019; Chou et al. 2016; Small and Laycock 2020; Foster et al. 2018; Horgas 2017; Ruano et al. 2022). Significant efforts are being directed towards optimizing these strategies, with ongoing studies aimed at identifying the most effective methods for pain management in the context of esophageal cancer surgery (Sunpaweravong et al. 2023).

Effective management of postoperative pain is essential, not only for enhancing patients’ comfort but also for mitigating associated complications, such as arrhythmias, which have recently emerged as an area of significant interest (Scheffer et al. 2017; Maeßen et al. 2023). Arrhythmias, particularly supraventricular tachycardia and atrial fibrillation, have been noted in postoperative patients and are believed to be linked to the physiological stress and pain experienced after surgery (Scheffer et al. 2017; Laferrière-Langlois et al. 2024). Understanding this relationship could prove crucial, as managing pain effectively might reduce the risk of arrhythmias and improve outcomes.

Nursing plays an integral role in the perioperative management of pain for ESCC patients (Luo et al. 2022). The ongoing care provided by the nursing team is essential for effective pain management, as they apply their professional expertise and extensive experience to deliver tailored and comprehensive pain relief strategies consistently (Poulsen and Coto 2018; Wang et al. 2020). Through their professional knowledge and rich experience, nursing staff offer personalized and comprehensive pain relief strategies for patients, constantly assessing the level and impact of pain (Zarzycka et al. 2019). Additionally, through effective communication and psychological support, the nursing staff aid patients in positively dealing with pain and recovery issues, enhancing their quality of life and satisfaction (Coyer et al. 2007). However, globally, research on perioperative pain management strategies for ESCC remains limited, lacking systematic and long-term comparative studies, and thus needs further investigation.

This study aims to systematically compare the effects of different perioperative pain management strategies on patients with esophageal squamous cell carcinoma (ESCC) through a retrospective cohort study. By utilizing data from patients treated surgically at the Affiliated Huai’an No. 1 People’s Hospital of Nanjing Medical University, we seek to evaluate the effectiveness of various pain management strategies during the perioperative period. This research provides scientific evidence for clinical practice and decision-making, intending to improve postoperative pain management and promote patient recovery.

## Materials and methods

### Study design and participants

**Fig. 1:**
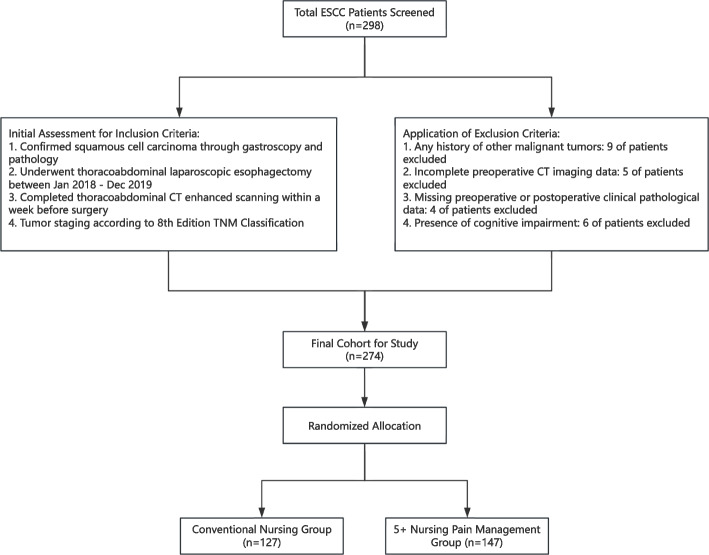
Flowchart of patient selection and group allocation in the study. From an initial screening of 298 esophageal squamous cell carcinoma (ESCC) patients, 274 were included in the final cohort after applying inclusion and exclusion criteria. The final cohort was randomized into two groups for the study: Conventional Nursing Group (*n* = 127) and 5 + Nursing Pain Management Group (*n* = 147)

This retrospective cohort study was designed to compare the effectiveness of different pain management strategies during the perioperative period of esophageal squamous cell carcinoma (ESCC). Participants were patients who underwent thoracoabdominal laparoscopic esophagectomy at the Department of Thoracic Surgery, the Affiliated Huai’an No. 1 People’s Hospital of Nanjing Medical University, between January 2018 and December 2019. Inclusion criteria were meticulously established to ensure a homogenous study population, including the following: (1) confirmed diagnosis of squamous cell carcinoma via gastroscopy and pathological examination, (2) completion of a thoracoabdominal CT-enhanced scanning within 1 week prior to surgery, and (3) tumor staging according to the 8th edition TNM classification, with documentation in the study records. To minimize selection bias and ensure the reliability of the findings, the following exclusion criteria were applied: (1) history of other malignant tumors, to eliminate confounding influences on outcome assessments; (2) the absence of preoperative CT imaging data, as such lack renders adequate treatment planning and baseline risk evaluations impossible; (3) incomplete preoperative and postoperative clinical pathological data, restricting comprehensive assessments of therapeutic outcomes; and (4) cognitive impairments diminishing patient ability to reliably perceive or communicate pain levels and other symptoms. From an initial pool of 298 patients meeting eligibility, a total of 274 patients were finally enrolled in the study, as illustrated in Fig. 1. Specifically, 127 patients received conventional nursing measures, whereas 147 patients were managed using the “5 + nursing” strategy. The implementation of this study will abide by the guidelines of the Declaration of Helsinki and is authorized by the Ethics Committee of Nanjing Medical University (KY-2024–114-01).

### Interventions and outcome indicators

Outcome observation indicators include analgesic doses (including average daily opioid use and average daily non-opioid medication use), postoperative pain score, and types of postoperative analgesic side effects.

The interventions in the control group of this study included the use of traditional non-opioid analgesics such as paracetamol (acetaminophen) and ibuprofen. The typical dosage for paracetamol was 1000 mg taken orally every 8 h, while ibuprofen was administered at 400 mg every 8 h as needed, based on patient-reported pain levels from the 1st to the 7th day post-surgery. For the optimized use of opioid drugs in the 5 + nursing group, medications such as tramadol and morphine were prescribed. Tramadol was administered at a dose of 50 mg orally every 6 h as needed for moderate pain. Morphine was reserved for more severe pain and administered intravenously, starting at a dose of 2–4 mg every 4–6 h, titrated to the minimum effective dose required for pain control. The average daily dose of opioids aimed to be maintained within the range of 37.5–50 mg in oral morphine equivalents to optimize pain management while minimizing potential side effects and risk of opioid dependence. Additionally, within the 5 + nursing strategy, the inclusion of physical therapy, rehabilitation measures, and methods such as cold compresses, hot compresses, massage, physiotherapy, psychological support, and music therapy aimed to holistically address the patients’ pain and overall recovery.

The postoperative pain score is measured once a day from the 1st to the 7th day after surgery. Postoperative pain scores use a visual analog scale (VAS) and a (Antony et al. 2019) FPS to score separately (Dourado et al. 2021; Chiarotto et al. 2019). The VAS score was recorded at fixed time points each day: morning (8:00 AM), afternoon (2:00 PM), and evening (8:00 PM). The FPS was recorded once daily, typically during the evening assessment, to accommodate patients’ cognitive preferences and ensure a comprehensive understanding of their pain levels. For the purpose of analysis, the data were processed in two different methods. First, we calculated the daily average VAS score by averaging the three time-point scores recorded each day. Secondly, for each day, we identified the minimum and maximum VAS scores, allowing us to capture variations in pain experiences throughout the day. The rationale for incorporating the FPS in addition to the VAS was to accommodate varying levels of literacy and cognitive ability among the patient population, thereby ensuring that all patients could effectively communicate their pain levels. The FPS, which uses facial expressions to represent different levels of pain, is an intuitive tool that is particularly beneficial for patients who may have difficulty using a numerical or abstract scale due to language barriers or cognitive challenges. Hence, the use of both VAS and FPS allowed for a more comprehensive and inclusive evaluation of postoperative pain, enhancing the accuracy and reliability of our pain assessment.

Common side effects monitored included nausea and constipation, which were documented as either present or absent, aiding in evaluating the tolerability of the analgesic regimen.

### Sample size calculation

The sample size for this retrospective cohort study was determined based on power analysis, ensuring adequate statistical power to detect meaningful differences in postoperative pain and related outcomes between different pain management strategies. Calculations were conducted using a priori power analysis, targeting a power of 80% and an alpha level of 0.05 to account for type I error. The anticipated effect size was derived from pilot data on postoperative pain reduction, with adjustments for potential confounders such as age, sex, and comorbid conditions—factors known to influence postoperative pain and recovery in ESCC patients.

### Statistical analysis

Continuous variables are presented as means and standard deviations if normally distributed; however, due to the potential non-normal distribution of clinical data (e.g., extreme values or skewness), the Kolmogorov–Smirnov test and Shapiro–Wilk test were utilized to confirm data normality. Nonparametric tests, including the Mann–Whitney *U*-test, were employed to assess differences between groups when normality assumptions were violated, providing reliable inference without being influenced by distributional abnormalities. This choice is crucial in maintaining the robustness and integrity of statistical conclusions, especially given the potential for skewed distributions in clinical measurements like pain scores or hospital stay durations. Categorical variables were reported as frequencies and percentages, and differences between groups were evaluated using the chi-square test. Multivariable logistic regression models were constructed to assess the impact of different pain management strategies on the occurrence of postoperative complications, incorporating stepwise selection methods based on Akaike’s information criterion (AIC) to optimize model variables. The results from logistic regression were expressed in terms of adjusted odds ratios (ORs) with 95% confidence intervals (CIs). All statistical analyses were conducted using free statistics software version 2.0 and the R software package (http://www.R-project.org, R Foundation). Differences were considered statistically significant when *P* < 0.05.

## Results

### Baseline characteristics

As shown in Table 1, a total of 274 ESCC patients were enrolled in the study. Of these, 127 patients received conventional nursing measures for pain management, and 147 received 5 + nursing measures for pain management. The baseline characteristics of the two groups of patients, such as gender, age, FEV1/FVC%, diabetes, hypertension, smoking, drinking, and tumor stage, had no significant differences, indicating that the two groups of patients are comparable. The results show that patients using the 5 + nursing measures for pain management had significantly lower postoperative VAS score, FPS score, and comprehensive pain score than the conventional nursing group, indicating that the 5 + nursing pain management strategy can effectively reduce the postoperative pain of patients. Notably, the average daily dose of non-opioid analgesics in the group of patients who used the 5 + nursing pain management measures was not significantly different from that in the conventional nursing group, showing that the pain control effect of the 5 + nursing is not dependent on the increased use of non-opioid analgesics. In observation of perioperative complications in patients, there was no significant difference in the incidence of postoperative analgesic side effects such as nausea and constipation between the two groups, indicating that while effectively controlling pain, the 5 + nursing pain management measures did not increase the patient’s postoperative analgesic side effects. In addition, patients using the 5 + nursing pain management measures had a significantly shorter length of hospital stays than those in the conventional nursing group, which may indicate that it helps improve the recovery speed and medical efficiency of patients.

**Table 1:** Classification and clinical characteristics of patients based on measures for pain relief

Variables	Total (*n* = 274)	Conventional nursing measures (*n* = 127)	5 + nursing measures (*n* = 147)	*p*-value
Gender, *n* (%)				0.901
Female	191 (69.7)	89 (70.1)	102 (69.4)	
Male	83 (30.3)	38 (29.9)	45 (30.6)	
Age, mean ± SD	64.9 ± 6.5	64.2 ± 6.7	65.5 ± 6.3	0.101
FEV1/FVC%, mean ± SD	88.7 ± 19.6	89.0 ± 19.4	88.4 ± 19.9	0.815
Diabetes mellitus, *n* (%)				0.176
No	186 (67.9)	81 (63.8)	105 (71.4)	
Yes	88 (32.1)	46 (36.2)	42 (28.6)	
Hypertension, *n* (%)				0.274
No	221 (80.7)	106 (83.5)	115 (78.2)	
Yes	53 (19.3)	21 (16.5)	32 (21.8)	
Smoking, *n* (%)				0.898
No	178 (65.0)	82 (64.6)	96 (65.3)	
Yes	96 (35.0)	45 (35.4)	51 (34.7)	
Drinking, *n* (%)				0.332
No	185 (67.5)	82 (64.6)	103 (70.1)	
Yes	89 (32.5)	45 (35.4)	44 (29.9)	
Prealbumin, mean ± SD	270.0 ± 20.7	264.4 ± 21.8	274.8 ± 18.5	< 0.001
T stage, *n* (%)				0.069
T1	75 (27.4)	41 (32.3)	34 (23.1)	
T2	71 (25.9)	36 (28.3)	35 (23.8)	
T3	128 (46.7)	50 (39.4)	78 (53.1)	
N stage, *n* (%)				0.285
N0	172 (62.8)	80 (63)	92 (62.6)	
N1	61 (22.3)	25 (19.7)	36 (24.5)	
N2	30 (10.9)	14 (11)	16 (10.9)	
N3	11 (4.0)	8 (6.3)	3 (2)	
Arrhythmia, *n* (%)				0.068
No	222 (81.0)	97 (76.4)	125 (85)	
Yes	52 (19.0)	30 (23.6)	22 (15)	
Pleural effusion, *n* (%)				0.09
No	252 (92.0)	113 (89)	139 (94.6)	
Yes	22 (8.0)	14 (11)	8 (5.4)	
VAS score, mean ± SD	6.0 ± 2.2	7.1 ± 2.3	5.0 ± 1.5	< 0.001
FPS score, mean ± SD	5.6 ± 2.0	6.7 ± 1.7	4.7 ± 1.7	< 0.001
Postoperative pain score, mean ± SD	5.8 ± 1.6	6.9 ± 1.5	4.9 ± 1.1	< 0.001
Daily non-opioid medication dosage, mean ± SD	813.1 ± 272.5	809.4 ± 280.4	816.3 ± 266.4	0.835
Daily opioid medication dosage, mean ± SD			44.9 ± 20.93	
Nausea, *n* (%)				0.308
No	233 (85.0)	111 (87.4)	122 (83)	
Yes	41 (15.0)	16 (12.6)	25 (17)	
Constipation, *n* (%)				0.252
No	256 (93.4)	121 (95.3)	135 (91.8)	
Yes	18 (6.6)	6 (4.7)	12 (8.2)	
Length of hospital stays, mean ± SD	15.6 ± 6.9	17.3 ± 6.8	14.1 ± 6.7	< 0.001

Note: The calculation method for the postoperative pain score is to take the average of the VAS score and FPS score

### Univariate and multivariate logistic regression analyses of postoperative arrhythmias

As seen from the results of the logistic multifactorial regression analysis in Table 2, postoperative pain score is independent risk factors for postoperative arrhythmias (adjusted *OR* = 1.27, 95% *CI* 1.04 ~ 1.54, *P* = 0.018). This result suggests that for every 1-point increase in postoperative pain score, the patient’s risk of postoperative arrhythmia increases by 0.27 times. Therefore, good perioperative pain control strategies may have a significant impact on the risk of postoperative arrhythmia in ESCC.

**Table 2:** Results of univariable/multivariable logistic regression analysis and predictors of arrhythmia

Variable	Arrhythmia			
	crude.OR 95% *CI*	crude.*p*-value	adj.OR 95% *CI*	adj.*p*-value
Gender				0.844
Female	Ref		Ref	
Male	1.03 (0.53 ~ 1.98)	0.934	0.93 (0.47 ~ 1.86)	
Age	1.01 (0.96 ~ 1.05)	0.795	1 (0.96 ~ 1.05)	0.885
FEV1/FVC%	1.01 (1 ~ 1.03)	0.161	1.01 (1 ~ 1.03)	0.089
Diabetes mellitus				0.198
No	Ref		Ref	
Yes	1.42 (0.76 ~ 2.65)	0.278	1.54 (0.8 ~ 2.99)	
Hypertension				0.445
No	Ref		Ref	
Yes	1.15 (0.55 ~ 2.42)	0.714	1.36 (0.62 ~ 2.96)	
Smoking				0.232
No	Ref		Ref	
Yes	1.08 (0.58 ~ 2.03)	0.801	1.76 (0.7 ~ 4.44)	
Drinking				0.217
No	Ref		Ref	
Yes	0.81 (0.42 ~ 1.57)	0.534	0.54 (0.2 ~ 1.44)	
Prealbumin	0.99 (0.98 ~ 1.01)	0.299	1 (0.98 ~ 1.01)	0.607
Length of stay	1.04 (1 ~ 1.08)	0.072	1.03 (0.99 ~ 1.07)	0.175
Pain score	1.27 (1.06 ~ 1.53)	0.011	1.27 (1.04 ~ 1.54)	0.018

### Curve-fitting analysis of the influence of daily opioid medication dosage on postoperative pain score

**Fig. 2:**
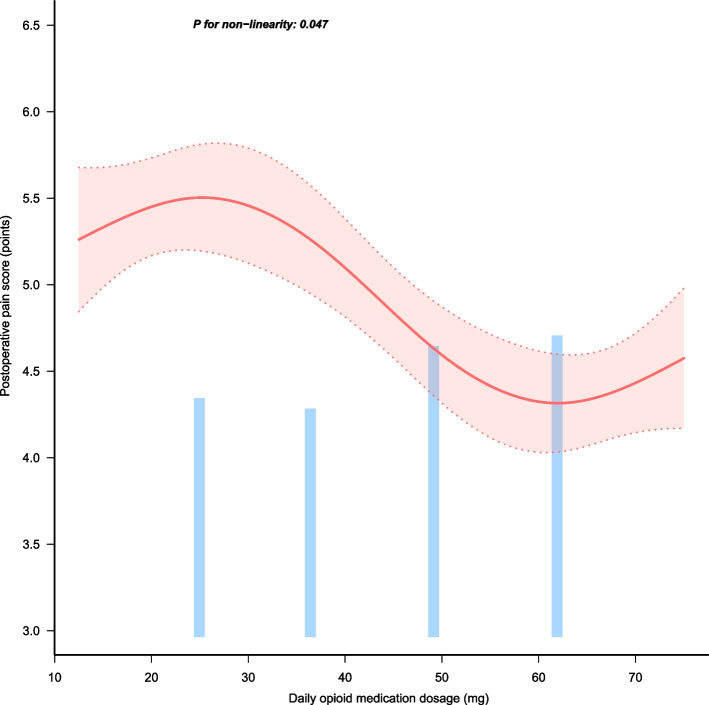
The figure depicts the results of curve-fitting analysis investigating the impact of daily opioid medication dosage on postoperative pain score. It reveals a nonlinear correlation, as indicated by a significant *p*-value for nonlinearity (*p* = 0.047). Notably, the patient’s postoperative pain score exhibits the steepest decline when the average daily opioid dosage ranges between 37.5 and 50 mg

As shown in Fig. 2, we can see that there is a nonlinear relationship between the average daily dose of opioids and the patient’s postoperative pain score (P for nonlinearity = 0.047). At the same time, when the average daily dose of opioids used is between 37.5 and 50 mg, the rate of decrease in the patient’s postoperative pain score is the fastest. This research result suggests that when using the 5 + nursing measures, it may be considered to control the average daily dose of opioids between 37.5 and 50 mg to achieve the purpose of quickly relieving the patient’s postoperative pain.

## Discussion

This retrospective cohort study took patients with esophageal squamous cell carcinoma who had undergone surgical treatment at the Affiliated Huai’an No. 1 People’s Hospital of Nanjing Medical University in recent years as research subjects and compared the effects of the regular care and the “5 + nursing” pain management measures during the perioperative period. The results showed that the postoperative pain level of patients using the “5 + nursing” management measures significantly decreased, and their hospital stays were significantly shorter compared to the regular group. Notably, there was no significant difference in the daily non-opioid analgesic dose between the patient group given the 5 + nursing pain management strategy and the regular care group, suggesting that superior pain control effects of 5 + nursing strategies are not dependent on an increased use of non-opioid analgesics. This offers a new and effective strategy for managing perioperative pain in esophageal squamous cell carcinoma patients.

The core objective of the “5 + nursing” strategy is to redefine and enhance the role of nursing in perioperative pain management by integrating multidisciplinary and comprehensive care modalities. This strategic approach involves not just managing the physiological aspects of pain but also encompassing a broader spectrum of patient care, prioritizing education, psychological support, and holistic rehabilitation measures (Sobczak and Goryński 2020; Singh et al. 2020; Prideaux and Marshall 1994; Yorkgitis et al. 2019; Humphrey and Malone 2015; van Melick et al. 2016). Unlike traditional nursing, which primarily focuses on basic care and routine monitoring, the “5 + nursing” approach positions nursing at the heart of an interdisciplinary framework. This framework extends beyond conventional practices by involving nurses actively in assessing, planning, and implementing diverse intervention strategies. In this context, nurses serve as the linchpin connecting patients with various therapeutic disciplines, involving them in personalized care pathways that are crucial for accelerated recovery and enhanced quality of life (Stamenkovic et al. 2018; Ustunel and Erden 2022). Incorporating elements such as optimal opioid use, preoperative education, physical therapy, and adjunctive therapies (e.g., cold compresses, massage, music therapy) and facilitating patient self-management empower patients to take an active role in their health care journey. This strategy not only optimizes physical outcomes but also significantly influences patients’ psychological resilience, combating anxiety and fostering positive attitudes towards recovery. By promoting education, nurses help demystify the surgical experience, equip patients with coping strategies, and enhance adherence to pain management regimens (Rekatsina et al. 2021; Myles et al. 2017).

However, the study also found that the pain score is an independent risk factor for postoperative arrhythmia, indicating that perioperative pain management has a significant impact on the risk of postoperative arrhythmia in patients with esophageal squamous cell carcinoma (Cronin et al. 2022; Karamchandani et al. 2020). This not only underlines the importance of pain management in controlling postoperative complications but also reflects that more research is needed to explore the potential mechanisms between perioperative pain management and postoperative complications.

Furthermore, our study found that the daily dose of non-opioid medication is negatively correlated with the postoperative pain score, and when the daily opioid dose is between 37.5 and 50 mg, the patient’s postoperative pain score drops the fastest (Waelkens et al. 2021). This suggests that we should consider finding the opioid dose that can maximally improve each patient’s postoperative pain during the perioperative period, enabling them to relieve pain better and speed up recovery.

In the realm of esophageal squamous cell carcinoma surgery, perioperative pain management is a cornerstone of patient care, and selecting the optimal strategy is critical for enhancing recovery and reducing complications. Current pain management strategies, which include the routine use of epidural analgesia, intravenous opioid administration, and non-opioid analgesics, are frequently integrated into patient care plans. However, each approach presents its own set of benefits and limitations, making the choice complex and often highly patient specific.

Epidural analgesia is traditionally employed for controlling acute postoperative pain due to its ability to deliver direct and potent analgesics to the epidural space, significantly reducing the need for systemic opioids (Jiang et al. 2025). Numerous studies have attested to the efficacy of epidural analgesia in reducing postoperative pain scores and improving patient satisfaction (Zang et al. 2025). Despite these advantages, epidural analgesia is not without risks—it may introduce complications such as hypotension, urinary retention, and the rare but severe epidural hematoma (Babu et al. 2024). Furthermore, its efficacy is sometimes limited by technical challenges or patient-specific contraindications.

Intravenous opioid administration, commonly utilized within the context of patient-controlled analgesia (PCA), offers an alternative that allows for better dosage titration tailored to individual pain thresholds and needs (Myers et al. 2024). The PCA method empowers patients to administer analgesics at their discretion, which can lead to enhanced perception of control over pain and overall satisfaction. Yet, concerns about potential opioid-related side effects—such as respiratory depression, nausea, and constipation—and the risk of developing dependence necessitate cautious administration (Altenau et al. 2017). Moreover, studies have shown that optimizing opioid dosages in concert with adjunct therapies can significantly mitigate these risks while preserving analgesic effectiveness (Dowell et al. 2022).

Non-opioid analgesics, including NSAIDs like ibuprofen and acetaminophen, are widely used as part of multimodal analgesia strategies to leverage their anti-inflammatory properties and opioid-sparing effects (Tan et al. 2020). While generally tolerated, these medications bear their own limitations, such as gastrointestinal side effects, renal impairment risks, and variable efficacy on severe postoperative pain, particularly in major surgeries like esophagectomy (Eccleston et al. 2017).

Our study contributes to this discourse by presenting evidence supporting a comprehensive “5 + nursing” strategy, which integrates opioid administration, non-opioid analgesic use, and supplementary measures such as preoperative education and physiotherapy, showing a notable reduction in postoperative pain levels without increasing the reliance on non-opioid analgesics. Importantly, we also observe that postoperative pain scores relate significantly to the risk of arrhythmias, while optimal opioid dosing (37.5–50 mg daily) corresponds with rapid pain score improvement.

Current research on pain management for ESCC remains limited, with most studies having constrained durations or lacking systemic approaches. The documented benefits of multimodal strategies highlight the necessity for in-depth exploration of more integrative approaches, which could address these complex pain landscapes more effectively. Future investigations should aim to clarify the long-term effects of these strategies and focus on stratified research that considers patient-specific factors such as psychological profile and comorbid conditions. Additionally, more robust multicenter studies are warranted to generalize findings and optimize care protocols, ultimately enhancing patient outcomes and quality of life during their recovery journey.

However, this study also has some limitations. First, although we used a retrospective cohort study, we could not entirely avoid the influence of selection bias. Second, data from a single center may present a center effect, and therefore, validation through multicenter prospective research may be necessary. Thirdly, the study’s short-term observation results do not reflect the long-term pain management effects for patients with esophageal squamous cell carcinoma. Lastly, we did not further analyze the impact of patients’ psychological states on the effectiveness of pain management, despite the potential significant influence psychological factors may have on patients’ perceptions of postoperative pain and recovery.

In summary, our research indicates that a comprehensive care and recovery support package for esophageal cancer surgery patients, including opioids and non-opioids, individualized preoperative education, and postoperative pain management, is significant for improving patients’ quality of life. Future research should focus on further optimization, promotion, and implementation of such pain management strategies.

## Conclusion

The 5 + nursing pain management measures can effectively alleviate postoperative pain in patients with esophageal squamous cell carcinoma without increasing the dose of non-opiate analgesics or causing side effects from postoperative pain management. It may play a positive role in shortening hospital stays and reducing the risk of postoperative arrhythmia. Simultaneously, when applying the 5 + nursing pain management strategy, keeping the daily opioid dose between 37.5 and 50 mg rapidly and effectively relieves postoperative pain in patients.

## Data Availability

The corresponding author can be contacted to request access to the datasets used and/or analyzed during the current study. Please reach out to the corresponding author for further information regarding the availability of the datasets.
